# Nomophobia: An emerging problem

**DOI:** 10.3126/nje.v13i3.58932

**Published:** 2023-12-23

**Authors:** Indrajit Banerjee, Jared Robinson, Abhishek Kashyap, Brijesh Sathian

**Affiliations:** 1-3 Sir Seewoosagur Ramgoolam Medical College, Belle Rive, Mauritius; 4 Geriatrics and long term care department, Rumailah Hospital, Hamad Medical Corporation, Doha, Qatar

## Background

The past 20 years have played host to some of the most rapid and widespread advancements in technology and mobile telecommunications in human history. Mobile cellular devices have rapidly developed from cumbersome, mono-use, and expensive devices to all-in-one, multi-use, and powerful pieces of hardware, with capabilities far greater than the expectations and imagination of Alexander Graham Bell [1]. Furthermore, the development of technology has made mobile phones accessible to every class of society, from the top down.

The age of users is a boundary that has not curbed the permeation of mobile cellular devices, with both the elderly and young alike being just as engrossed in the use thereof. Never in the history of humankind has man been more reliant on such a piece of technology to fulfil his everyday needs, workings, and life. The great reliance on mobile devices and the increased time spent on them in the past two decades have brought about a host of newly classified medical conditions and diseases directly linked to their use, which were non-existent to the medical fraternity a few years ago. One such recently identified psychiatric condition is “nomophobia” or “no mobile phone phobia,” which is effectively the fear or phobia of being separated or detached from one’s mobile phone or the connectivity thereof [2].

### Origins of nomophobia

The term “nomophobia” was initially coined in a 2008 British postal service study [3]. Nomophobia is effectively the anxiety and fear that are induced when individuals are detached from their mobile phone; it is not only exclusive to being detached from the mobile device but also includes a lack of battery and internet connectivity or switching the device off. A 2008 study commissioned by the British postal service revealed that 53% of the ± 2000 participants suffered from “nomophobia.” The study also revealed that male participants suffered from nomophobia at a slightly higher rate than their female counterparts (58% and 47%, respectively) [4].

Nomophobia as a term, in its own right, is a misnomer, as “phobia” indicates a predilection to fear, whereas nomophobia is more commonly found to be an anxiety-type disorder. Nomophobia has, however, been described in the DSM-IV criteria and has been placed under the category of “phobia for particular or specific things.” As described in the introduction, the global use of mobile devices has exponentially increased since 2008, and thus, there is a global burden of disorders such as nomophobia and its counterparts. There is also a section of psychiatrists and physicians who are trying to get nomophobia explicitly and separately classified in the DSM-V [5, 6].

### Risk factors for nomophobia

Various risk factors are believed to contribute to the development of nomophobia. Individuals with low self-esteem, anxiety disorders, social disorders, general anxiety disorder, predilections to addiction (gambling addiction), or those who have experienced a traumatic event and could not call for aid due to the lack or absence of their mobile device may develop nomophobia [7].

### Signs and symptoms

The symptom cluster associated with “nomophobia” closely mimics that of anxiety disorders. Patients often present with one or more symptoms of sweating, hyperventilation, anxiousness, increased arousal, disorientation, and palpitations [7].

Some of the indicators of nomophobia are as follows: (1) the inability to turn the mobile device off; the mobile device remains switched on for 24 hours, 7 days a week. (2) Ability to be physically separated from the mobile device. (3) Concern about not being able to call for help without access to the device. (4) Lack of checking the phone and messages caused upset or anxiety. (5) The phone is constantly on the person, even in showers and bathrooms. (6) Constant verification of mobile device location [7].

### Prevention

As with most diseases, prevention is the best treatment option. In the case of mobile devices, the prevention of overuse is difficult and nearly impossible to control. Naturally, however, the increased use of the device increases the need for it on a daily basis; therefore, reducing its use and limiting screen time should be the first-line method of prevention. No legislature limits the duration of mobile device usage, and it is unlikely that such a legislature will ever come into fruition. Therefore, it is solely in the hands of the user or the parents of younger users to exercise self-control. The ban on mobile phones in schools and learning institutions is a simple yet effective method of prevention [8].

A new group of mobile applications has been developed to help combat the overuse of such devices, with the ability for the user to set daily usage limits in hours and thereafter lock the recipient out of various other applications on the device. Parental control applications have also become more popular and give parents the ability to not only control what content their children are viewing on such devices but also allow for access control and duration control. The technological company “Apple” has also recently built in monitors of screen time, which send users weekly reports of their screen time and mobile phone usage [9].

### Treatment

The treatment of nomophobia includes both pharmacological and nonpharmacological methods. Non-pharmacological methods include cognitive behavioural therapy (CBT), stress reduction exercises, counselling, reduction of use via weaning off the device, and relaxation techniques. Pharmacological therapies include the administration of psychotropic drugs, most commonly in the SSRI-selective serotonin reuptake inhibitor group [10].

### Conclusion

The rapid rise in the use of and reliance of the human race on technological devices such as mobile phones in the 21st century has precipitated nomophobia, and the current usage of such devices is only on the incline. The global burden of nomophobia will continue to rise, and it is therefore prudent that the public be better educated about nomophobia and their devices, as well as the negative side effects of the overuse thereof. Prevention by limiting screen time and taking self-control measures remains the best approach to curbing precipitation and the development of nomophobia.
